# Effect of Freezing Wheat Dough Enriched with Calcium Salts with/without Inulin on Bread Quality

**DOI:** 10.3390/foods11131866

**Published:** 2022-06-24

**Authors:** Angela Daniela Carboni, Andrea Gómez-Zavaglia, Maria Cecilia Puppo, María Victoria Salinas

**Affiliations:** 1 Centro de Investigación y Desarrollo en Criotecnología de Alimentos (CIDCA), Facultad de Ciencias Exactas, Universidad Nacional de La Plata (UNLP)-CONICET, 47 y 116, La Plata RA1900, Argentina; angela.carboni@hotmail.com (A.D.C.); mcpuppo@quimica.unlp.edu.ar (M.C.P.); 2 Facultad de Ciencias Agrarias y Forestales, Universidad Nacional de La Plata, 60 y 122, La Plata RA1900, Argentina

**Keywords:** prebiotics, calcium, frozen dough, fortified breads, bread quality

## Abstract

Bread is a popular food that is widely consumed worldwide but has a short shelf life. Besides that, when incorporating prebiotics and calcium, aging mechanisms accelerate, further shortening the shelf-life. The objective of this work was to evaluate the effect of freezing storage on the rheological (loss tangent, tan δ) and thermal (glass transition temperature, Tg) properties of unfrozen dough, the fermentation times (tf), and the baking quality of wheat bread fortified with calcium and inulin. Formulations studied included wheat flour (control-C), flour with 1800 ppm Ca (calcium carbonate-CA, calcium citrate-CI or calcium lactate-LA), and flour with 2400 ppm Ca and 12% inulin (calcium carbonate-CA-In, calcium citrate-CI-In or calcium lactate-LA-In). Doughs were stored at −18 °C for 1, 7, 30 and 60 days. After storage, the rheological (oscillatory rheometry and texture profile analysis) and thermomechanical properties of the thawed doughs were measured. The quality parameters of breads determined consisted of specific volume (Vs), color, moisture, firmness, elasticity, and alveoli size characterization. Dough freezing neither changed viscoelasticity (tan δ) nor decreased hardness and adhesiveness up to the values observed for fresh wheat dough. The Tg of dough with calcium carbonate increased, while for samples with organic calcium salts, it (citrate and lactate) decreased. The tf of thawed dough significantly increased. The Vs of all breads did not change during the first 30 days but decreased after freezing the dough for 60 days (*p* < 0.05), probably due to the death of the yeasts. Crumb moisture decreased over time, and in all cases crumb C had the highest moisture content, suggesting a dehydration effect of the calcium salt. The firmness of CA, LA and C crumbs were similar and higher than that of CI (*p* < 0.05), suggesting a destabilizing effect of CI anion on gluten proteins. Inulin contributed to the depreciation of bread quality, mainly at 60 days of dough freezing storage. It can be concluded that during freezing storage, calcium improves the dynamic elasticity of the dough, although under extreme conditions it generates loaves of smaller volume. Principal component analysis (PCA) explained 66.5% of total variance. Principal component 1 (PC1) was associated with dough properties, and accounted for 44.8% of the total variance. In turn, PC2 was mainly related to baking quality parameters (fermentation time, browning index, firmness and springiness of crumbs), and explained 21.7% of the total variance. Fortification with calcium citrate should be recommended for dough freezing, as breads with softer crumbs were obtained under such conditions.

## 1. Introduction

Fresh bread has a short shelf life, because different physical and chemical changes occur during storage, known as bread aging. During this stage, the crumb hardens, there is a loss of cohesiveness, and the characteristic aroma of freshly baked bread is lost, among other changes. The process begins immediately after baking. Starch reorganizes, leading to a certain crystallinity [1,2]. Hardening occurs as a result of amylopectin retrogradation, with the loss of crumb elasticity and crust softening because of the water migration from the crumb to the surface [1].

The freezing process of dough avoids the large economic losses usually caused by the aging of baked products and offers to the consumer the possibility of having a freshly prepared product at any time [3]. A liquid phase (free water) dispersed in a predominantly amorphous solid matrix can be identified in bread dough, with any remaining crystalline structures arising from the structural organization of starch molecules; yeasts are also dispersed. During freezing, water is separated as ice, whereas gluten slowly dehydrates. The damage of yeasts occurs during the freezing process and is dependent on the conditions and time of storage in the frozen state. The weakening of the dough during such storage may arise from a loss of the degree of cross-linking of the gluten network, associated to different processes, including the mechanical action of ice crystals, a redistribution of water or the release of reducing compounds by yeasts during freezing [4]. Varriano-Marston et al. [5] proposed that ice contributes to the weakening of the gluten network, and part of this protein network is cleaved by the mechanical action of the crystals. These authors also propose other mechanisms for dough weakening as water redistribution due to ice formation and recrystallization during freezing and frozen storage.

During thawing water crystals melt; however, water does not return to its original state and place in the gluten matrix. Gluten dehydration has negative effects on the dough structure and the quality of baked products [6]. Although several causes were proposed for explaining dough weakening, changes are most likely due to a combination of all of them.

Food components in dough and baked goods may exist in either crystalline, partially crystalline or amorphous states, all of them contributing to the product’s properties and stability [7]. In addition to the major components of starch and gluten, both of which are commonly found in the amorphous state in frozen dough, several other components can also alter the glass transition of bakery products, including water, oil, sugar, salt, and other additives like inulin [8]. A way of analyzing changes in frozen dough during thawing is through the evaluation of the glass transition temperature (Tg). This temperature reflects the gradual and reversible transition from a glassy (crystalline) to a rubbery (amorphous) state of a system and can be evaluated by Dynamic Mechanical Analysis (DMA), which determines the thermomechanical properties of viscoelastic materials as a function of time, temperature or frequency [9].

Many studies have reported that the addition of inulin to flour improves the quality of bread [10]. Inulin is a stable, healthy, and edible ingredient. It is a polysaccharide acting as a soluble dietary fiber and is constituted by molecules of fructose linked by β (2/1) glycosidic bonds, with a terminal glucose unit linked by a (1/2) bond. It has a degree of polymerization (DP) ranging from 2 to 60. According to the DP, inulin can be divided into short-chain (DP ≤ 10) and long-chain inulin (DP ≥ 23) [11].

The healthy benefits of inulin are largely related to its chemical structure because the chemical bonds are not hydrolyzed by human digestive enzymes in the small intestine, remaining intact until it reaches the colon, where it is totally hydrolyzed and fermented by bifidobacteria and lactobacilli [12]. For this reason, inulin is considered as a prebiotic, largely employed as a healthy food ingredient. However, the interest in this polysaccharide is associated with its technological functions as a stabilizer and fat substitute, and that it improves the texture of baked and other food products [13]. Inulin changes the rheological properties, viscosity, and hardness of dough, as it restricts the mobility of water and affects the taste properties of the products [14,15]. Ke et al. [16] studied the effects of inulin on proteins in frozen dough, finding that inulin (especially those of high degree polymerization) can protect proteins of dough during the storage freezing process. This behavior was attributed to a good combination of inulin and water, thus weakening the formation and recrystallization of ice crystals in the frozen dough as a consequence of inulin improving the quality of frozen dough. In addition, the damage to the disulfide bonds and hydrogen bonds were reduced in the gluten protein in the dough with the prebiotic.

On the other hand, refined wheat flour has fewer minerals and vitamins due to the milling process, decreasing its nutritional value. This effect can be compensated by fortifying the flour with mineral salts containing different cations such as calcium, magnesium, and iron [17]. A diet with adequate calcium intake prevents osteopenia and osteoporosis [18]. In addition, it is necessary to increase their bioavailability. In this sense, Salinas et al. [19] found a positive effect of inulin on the absorption and mineralization of calcium in male *Wistar* rats when they were fed with breads containing calcium carbonate and inulin. These authors also found a positive effect on the intestinal microbiota favoring the development of healthy microorganisms which was ascribable to the presence of inulin.

Previously, Salinas et al. [20] studied the effect of wheat flour enriched with different calcium types and inulin on hydration and rheological properties of dough, and the optimization of the formulation of the nutritional bread [21]. Although baked products containing calcium and inulin present a high nutritional value, these ingredients increase the aging processes of the bread [22]; therefore, it is necessary to implement a technique that improves the shelf life of baked products, such as dough freezing. Consequently, the objective of this work was to evaluate the rheological and mechanical properties of the dough after storage at −18 °C and the final baking quality of breads fortified with different calcium salts and inulin prepared with those frozen and thawed dough.

## 2. Materials and Methods

### 2.1. Materials

A commercial wheat flour (Molino Campodónico Ltd., La Plata, Argentina) type 0000 in accordance with Argentinean Alimentarius Codex [23] for breadmaking (9.92% proteins, 0.86% lipids, 0.382% ash, 11.8% moisture) was used. Farinographic parameters of this flour were: water absorption = 57.9 mL, development time = 18 min, stability = 38 min and, softening degree = 12 BU. The alveographic parameters were: tenacity-P = 132 mm, extensibility-L = 47 mm, and work-W = 264.

Prebiotic. A combination of inulin of high (30%) and low (70%) degree of polymerization (DP) (Synergy 1^®^, BENEO Orafti, Oreye, Belgium, 92.7% db) was used to achieve beneficial physiological effects.

Calcium. Different calcium salts were employed, namely calcium carbonate (CaCO_3_, ANEDRA S.A, Los Troncos del Talar, Buenos Aires, Argentina), calcium citrate [Ca_3_(C_6_H_5_O_7_)_2_.4H_2_O, Sigma-Aldrich (Burlington, MA, USA)] and calcium lactate [Ca(C_3_H_5_O_3_)_2_, Sigma-Aldrich)].

Other ingredients included sodium chloride (Celusal, Vicente López, Buenos Aires, Argentina), fresh yeast (CALSA, Lanús, Buenos Aires, Argentina), and distilled water.

### 2.2. Formulations and Preparations of the Dough

Mixtures containing wheat flour, 2% NaCl (wheat flour basis, wfb), fresh yeast (3% wfb), and calcium salt and inulin were prepared using the factor levels proposed by Salinas et al. [20] and Salinas and Puppo [21] (Table 1). In a previous work [20,21], where technological aspects of fresh dough were studied, all points of the central composite design were analyzed. In this work, only two points were selected for studying the freezing technology of dough: dough with the maximum level of calcium without inulin and the sample with the same amount of calcium salt and the highest level of inulin. Farinographic parameters (water absorption and development time) of the different blends are shown in Table 1. A wheat control (C) sample without calcium or inulin was studied.

Solid ingredients (wheat flour, salt, calcium salt, inulin) were first mixed for one minute in a planetary mixer (Kenwood Major, Havant, UK), then an optimal quantity of water (W_abs_) was added and kneaded during the development time (t_d_). For the first minute it was kneaded at 50 rpm and the rest of the time it was kneaded at 90 rpm until the development time was completed. The obtained dough was laminated (four passes) in a manual laminator (0.5 cm thick), left to rest at room temperature (15 min), covered with film to prevent surface dehydration. For rheological and thermomechanical analysis, dough without fresh yeast were prepared, while for the fermented doughs the yeasts were dispersed in the water and was added into solids.

### 2.3. Dough Freezing Process

Different fractions of dough without yeast (50 g) that were individually wrapped in film and placed in a Ziploc bag were stored in the freezer (Gafa, Rosario, Santa Fe, Argentina) at −18 °C for 0, 1, 7, 30 and 60 days. Once the storage time was over, dough portions were placed into a fermenter (Brito Hnos, Los Polvorines, Buenos Aires, Argentina) at 30 °C and 70% RH (relative humidity) for two hours until thawing.

### 2.4. Hydration, Rheological and Thermomechanical Properties of Dough

#### 2.4.1. Moisture and Water Activity

Moisture content of dough was determined according to the AACC method 44-19 [24] using a stove, (San Jor, Buenos Aires, Argentina) and water activity was measured with an equipment meter, an Aqualab series 3 (Decagon Devices Inc., Washington, WA, USA). Values correspond to the average of three determinations in both parameters.

#### 2.4.2. Dough Texture

Dough was laminated (1 cm in height) before cutting. Cylindrical dough pieces (diameter = 30 mm) cut with a cylindrical aluminum puncher were obtained for assays. The dough texture was analyzed performing a Texture Profile Analysis using a TA.XT2i Texture Analyzer (STABLE MICRO SYSTEMS, Surrey, UK) with a load cell of 25 kg and Texture Expert for Windows version 1.2 Software. Dough samples (*n* = 15) were utilized. Each sample was subjected to two cycles of compression (deformation = 40%, crosshead speed = 0.5 mm/s) with a cylindrical probe (P/75). Hardness (Hard), adhesiveness (Adh), and springiness (Sprin) of dough were calculated. Assays were performed in duplicate.

#### 2.4.3. Dough Viscoelasticity

Measurements were performed in a Haake RS600 (Thermoelectron, Karlsruhe, Germany) at 25 ± 0.1 °C using a plate-plate serrated surface sensor system (35 mm diameter) with a 1.5 mm gap between plates. The upper plate was lowered, and the excess of sample was trimmed off. The exposed surface was covered with a thin layer of semisolid silicone to prevent moisture loss during testing. Samples were rested for 15 min for relaxing before testing. Frequency sweeps (from 0.005 to 100 Hz) at constant deformation (5 Pa) within the linear viscoelastic range were performed. Mechanical spectra were obtained by recording the dynamic moduli G′, G″ and tan δ as a function of frequency.

#### 2.4.4. Dynamic Mechanical Analysis (DMA) of Dough

A dynamic mechanical analyzer (Q800 Dynamic Mechanical Analyzer, TA Instruments, New Castle, DE, USA) was used. During dynamic heating, the samples were analyzed at frequencies of 1, 3, 5, 7 and 10 Hz and the amplitude was 7 μm. Fresh dough was included in acrylic bars (60 mm × 13 mm × 4 mm) which were wrapped with a polyethylene film (to prevent water loss during the test) and placed in liquid nitrogen. After those molds were rested in the freezer (∼6 h) to induce maximal freeze concentration and this sample will be considered as the 0-day time for testing; molded dough was also rested up to 60 days before DMA analysis. The film was removed before mechanical assay and samples were loaded into the dual cantilever clamp; the screws were finger-tightened and the furnace head snapped into place. Then, samples were cooled and kept at −100 °C for 10 min and then heated to 40 °C at 3 °C/min. Storage modulus (E′), loss modulus (E″) and the loss tangent (tan delta) were recorded. Temperatures of glass transitions (Tg) and β-relaxation (Tβ) were obtained using the maximum peak of the loss modulus. Each measurement was performed at least in duplicate.

### 2.5. Breadmaking Process

#### 2.5.1. Fermentation Time Optimization

A portion of 50 g of the dough frozen with yeast and thawed for 2 h was placed into a graduated cylinder (500 mL) with a plunger mobile and inserted in a fermentation cabinet (Brito Hnos, Los Polvorines, Buenos Aires, Argentina) at 30 °C for 4 h and the increase in volume (ΔV) was registered as a function of time. The fermentation curves were adjusted by the Chapman model using the Sigmaplot 10.0 Software (www.systatsoftware.com).

(1) $$\Delta V=V_{\max}{\left[1-\exp(-\mathrm{bt})\right]}^{c}\;$$

where ΔV is the volume increment, t is the time and V_max_ (mL) corresponds to the maximum volume increment achieved. Parameters *b* and *c* are constant: *b* is the constant rate of dough fermentation (min^−1^) and *c* is related to the inflection point and the shape of the curve at the beginning of fermentation.

Fermentation time (t_f_) is the time required for achieving 3/4 of V_max_, because during the beginning of the baking process the fermentation continues until the structure is established.

#### 2.5.2. Baking Process

Dough with fresh yeast was prepared and 90 g portions were cut and rolled, left to rest for 10 min, and then French bread-type pieces were assembled in a bread maker (MPZ, Argentina). These pieces were frozen at −18 °C for 0, 1, 7, 30 and 60 days and thawing (2 h). They were then proofed at 30 °C according to their t_f_ and baked (26 min at 210 °C) in a convection oven (Ariston, La Tablada, Buenos Aires, Argentina). The quality of the bread was evaluated 2 h after baking. An assay was performed in duplicate.

#### 2.5.3. Bread Quality Evaluation

##### Bread Specific Volume

Specific volume (Vs) was determined according to AACC [24] as a ratio of volume and weight. Assays were performed in duplicate (*n* = 4 bread per formulation).

##### Crust Color

Color measurements were performed on the bread crust (*n* = 40 per formulation) using a tristimulus color analyzer (Chroma Meter CR 400, Konica Minolta, Osaka, Japan). Values of L*, a* and b* were measured and the browning index (BI) was calculated [15] according to Equations (2) and (3):

(2) $$\mathrm{BI}=\frac{100\left(X-0.31\right)}{0.172}$$

(3) $$X=\frac{a*+1.75L*}{5.645L*+a*-3.012b*}$$

##### Crumb Firmness

A texture profile analysis (TPA) of bread slices was performed using a texture analyzer TA.XT2i (Stable Micro Systems, Surrey, UK) equipped with a 25 kg load cell. From the middle part of each bread loaf, two slices of 2 cm height were obtained. A slice was subjected to a double compression cycle (deformation: 40%, crosshead speed: 0.5 mm/s) with a cylindrical probe (diameter = 2.5 cm), and firmness was determined. Eight replicates were analyzed for formulation.

##### Crumb Moisture

Moisture was determined employing the AACC method 44-19 [24]. Values obtained were the mean of three replicates.

##### Crumb Image Analysis

Crumb grain characteristics were analyzed by a digital image analysis system. Slices of the middle part of the loaves were obtained and scanned (138 dots per inch) with an HP Scanjet 4070 scanner. ImageJ software (Wayne Rasband, National Institute of Health, Kensington, MD, USA) was used in order to process the images. Squares of 2 cm × 2 cm (276 pixels × 276 pixels) of the scanned slices were converted into 8 bit images. Finally, a threshold was used to binarize the images, applying the IsoData algorithm (umbral = 209). The number of alveoli per area (N) and air fraction (AT) was defined as a percentage of the area occupied by the alveoli over the total area of the image, and the mean area of alveoli (AMM), were evaluated. Eight crumbs per formulation were evaluated.

### 2.6. Statistical Analysis

Statistical differences between groups were determined by a two-way analysis of variance using Infostat software, [25] followed by Fisher (LSD) to test whether the relationship between the two data sets was statistically significant (*p* < 0.05). A principal component analysis (PCA) was carried out to ascertain correlation between the dough properties and bread quality for the different calcium-inulin formulations using Infostat software (version 2018).

## 3. Results and Discussion

The freezing of wheat dough with prebiotics (inulin enriched in fructo-oligosaccharides) and fortified with different calcium salts (carbonate, citrate, and lactate) could affect the rheological and thermomechanical behavior of dough and the baking quality of breads made with dough after freezing.

### 3.1. Effects of Freezing Technology in Wheat Dough

**Figure 1:**
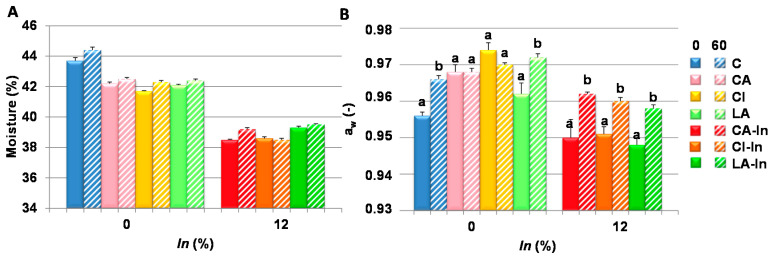
Moisture (**A**) and water activity (a_w_) (**B**) of the fresh and stored dough (−18 °C-60 days). Different letters indicate significant differences in the same formulation between 0 and 60 days (*p* < 0.05). Bars: mean ± standard deviation; (-) unitless parameter.

The moisture and water activity of the different fresh doughs (time 0) and those stored at −18 °C for 60 days are shown in Figure 1. The moisture of the wheat dough (C) was higher than that of the dough with calcium (CA, CI, LA). The lowest values were those of doughs with calcium (CA-In, CI-In, LA-In) and the prebiotic (Figure 1A). Water content did not significantly change during freezing, unlike water availability, and increased after storage in all doughs except in the CA and CI doughs (Figure 1B). During storage, although there was no dehydration of the doughs, the water availability changed after freezing, probably as a consequence of a structural change of the matrix due to the effect of freezing, thus leading to the greater availability of water. These changes could affect the viscoelastic characteristics of the dough. In this sense, Angioloni et al. [26] investigated the influence of freezing and storage time on dough’s viscoelastic performance, finding a lower positive viscoelastic performance of the dough, especially in cookies making weak wheat flours. This was attributed to the damage on the gluten cross-linking which was mainly produced by ice crystallization.

### 3.2. Rheological and Mechanical Behavior of Frozen Dough

The effect of freezing during low-temperature storage on dough rheology was evaluated by texture profile analysis (TPA), and in the range of linear viscoelasticity by oscillatory rheometry of the thawed dough. In addition, the thermomechanical properties were studied by dynamic mechanical analysis (DMA).

**Figure 2:**
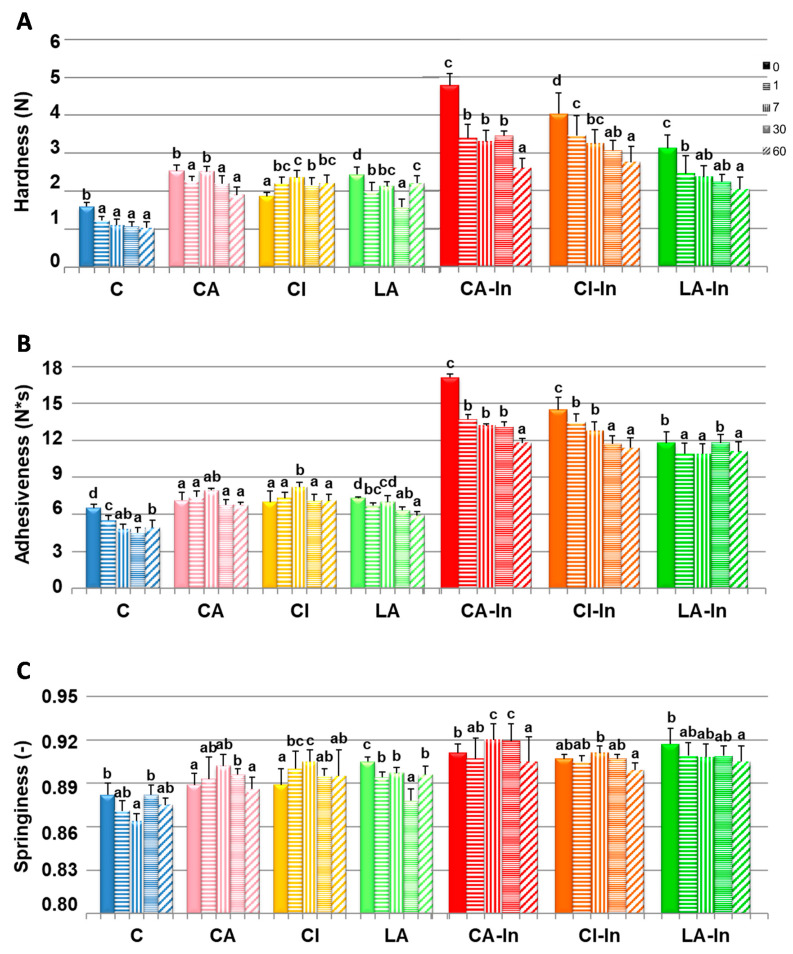
Textural parameters of doughs: Hardness (**A**), adhesiveness (**B**), and springiness (**C**) of the fresh and stored dough at −18 °C for 1, 7, 30 and 60 days. Different letters in the same formulation between 0 and 60 days of storage indicate significant differences (*p* < 0.05). Bars: mean ± standard deviation; (-): unitless parameter.

The parameters obtained through the TPA of the thawed doughs were hardness, adhesiveness, and springiness (Figure 2). At the same storage time, the wheat dough (C) was the softest; the addition of calcium and mainly inulin increased hardness, especially in samples with the prebiotic (CA-In and CI-In) (Figure 2A). The hardness of all the doughs decreased significantly after 60 days of storage, except for the dough with citrate without inulin (CI), which increased. A lower hardness during storage would be related to a weakening of the dough due to a redistribution of water caused by ice formation and recrystallization during freezing and frozen storage [4]. Several authors reported the effect of calcium lactate on different properties of wheat flours, such as modifications of the ionic strength of the dough [27]. The lactate anion destabilizes protein structure and together with inulin favors the formation of a less elastic gluten network [28]. A minor decrease in hardness in doughs with lactate and inulin (LA-In) with storage suggests that the prebiotic protects the dough from the destabilizing effects of the lactate ion.

The adhesiveness of the doughs is shown in Figure 2B. It was observed that for a given storage time, the wheat dough (C) was the least adhesive; the addition of calcium salts together with inulin increased this parameter (Figure 2B). After 60 days of freezing, the adhesiveness of C and LA doughs were significantly lower, whereas those of CA and CI doughs were statistically similar. In the presence of inulin, Ca-In and CI-In decreased the adhesiveness of the dough, while this property did not change in LA-In (Figure 2B).

In turn, at a given storage time, the springiness showed a dependence on the type of formulation (Figure 2C). Doughs with calcium lactate (LA and LA-In) presented a significant decrease in elasticity after 60 days of frozen storage.

**Figure 3:**
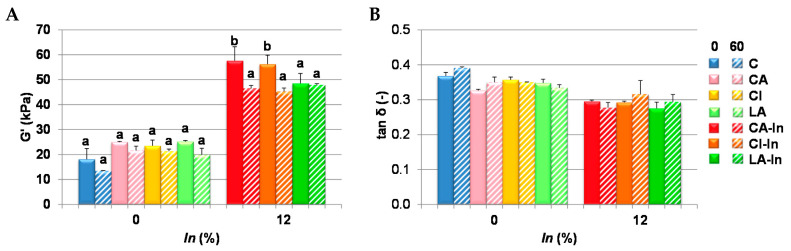
Rheological properties. (**A**) Elastic modulus (G′) and (**B**) loss tangent (tan δ) of fresh and stored doughs at −18 °C for 60 days. Different letters in the same formulation indicate significant differences (*p* < 0.05). Bars: mean ± standard deviation; (-): unitless parameter.

In the dynamic rheological test, the elastic modulus (G′) at a frequency of 1 Hz and the tan δ that relates the elastic and viscous moduli (tan δ = G″/G′) were determined under linear viscoelasticity conditions, this last parameter giving a more global idea of the viscoelastic behavior of the doughs. Figure 3 shows the G′ and tan δ of fresh (0 day) and thawed dough at the end of the storage period (60 days). The wheat dough (C) presented the lowest elastic modulus. The doughs treated at intermediate times gave similar results than those obtained with 60 days of freezing (data not shown). The doughs with calcium and inulin (CA-In, CI-In, LA-In) presented higher G′ than those prepared in the absence of inulin (CA, CI, LA).

The value of G′ did not change during storage in the doughs without inulin. This parameter significantly decreased with the storage time in doughs containing carbonate and citrate and inulin (CA-In and CI-In), and remained unaltered in doughs with lactate (LA-In). This suggests a protective effect of lactate anion against matrix de-structuring. No significant differences were found in tan δ after storage for any of the doughs studied (Figure 3B), therefore freezing did not produce changes in dough viscoelasticity after storage for 60 days at −18 °C. Similar results were found in dough with strong breadmaking wheat flours [26].

**Figure 4:**
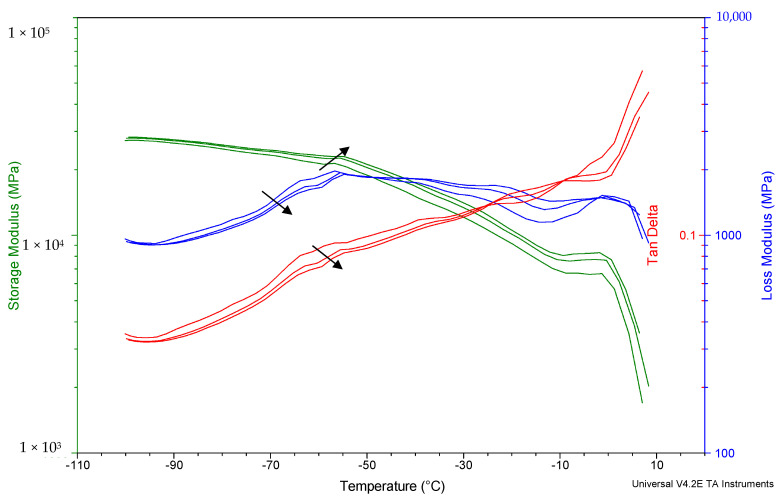
Storage modulus (E′), loss modulus (E″) and tan δ of wheat dough at 1, 5, 10 Hz obtained by DMA. The arrow goes from low (1 Hz) to high frequencies (10 Hz).

The thermomechanical properties of dough were studied by DMA analysis. Figure 4 shows the storage modulus (E′), loss modulus (E″) and tan δ of a typical thermogram obtained from a wheat dough. Four peaks in loss modulus (blue) and tan δ (red) curves were identified; each curve belongs to frequencies of 1, 5 and 10 Hz. Two higher peaks around −1 °C and −20 °C was observed, corresponding to the melting of ice and the start of ice melting, respectively [8]. The third peak, around—40 °C, corresponded to the α-relaxation (glass transition), while the lower temperature peak, at almost—60 °C, corresponds to a β-relaxation [9]. The temperatures of the different transitions increased with frequency. The frequency dependence behavior of transitions in bakery products using DMA has been reported in different studies [9,29].

Table 2 shows the values of temperatures of glass transition (Tg) and β relaxation (Tβ) of the different formulations of the fresh and stored (−18 °C for 60 days) doughs. The Tg of the non-stored wheat dough was −39.2 °C and was statistically similar to those of doughs with calcium citrate (CI-In, CI) and with lactate without inulin (LA). Doughs with carbonate (CA, CA-In) presented the lowest Tg, suggesting an amorphous state at temperatures above −50 °C; this result suggests that for this dough the polymers (proteins and starch) present high mobility, leading to a softer dough. On the other hand, LA-In presented the highest Tg, suggesting that calcium lactate induced a glass state in dough, and that molecules are able to move and go from the vitreous to the amorphous state at −34 °C. After 60 days of freezing storage, Tg measured at day 0 was statistically similar in the C (~50 °C) and CI doughs (~50 °C), while Tg increased with storage time in LA samples, but mainly in dough with carbonate (CA: −32 °C, CA-In: −39 °C). This increase indicates that regardless of the presence of inulin, calcium carbonate significantly modifies the gluten matrix crosslinking during storage, leading to a structure with a tendency to move to the rubbery state at higher temperatures. The opposite behavior was observed for doughs with organic calcium salts and inulin (CI-In, LA-In), for which Tg significantly decreased with storage; this effect was associated with a laxer or softer structure as a result of freezing, and was favored by inulin. This behavior could be due to the kind of interactions between inulin and these salts in the gluten matrix, as well as their capacity to hold water during freezing.

Meziani et al. [30] studied the effects of freezing treatments on the viscoelastic and structural behavior of frozen sweet dough (20% of sugar). They found no significant differences either in G′ or in tan δ in fresh and frozen dough at −20 °C. However, they reported a Tg value of −37 °C (measured by differential scanning calorimetry), in the same range as those obtained in this work. In turn, Ribotta et al. [9] stated that a shift of α-transition to a higher temperature in dough might be related to an increment of inter and intramolecular interactions, leading to an aggregated solid.

On the other hand, the amount of plasticizing unfrozen water affects the glass transition of freeze-concentrated systems [8]. In this sense, increasing the quantity of unfrozen water in the frozen concentrate, where the unfrozen water acts as a plasticizer, decreases Tg as a result of the increment of extensibility and the flexibility of the matrix [29]. As these changes in the glass transition temperature occurred at T < −18 °C (Table 2), at this storage temperature doughs are in an amorphous state.

At temperatures lower than Tg, there are secondary relaxations, such as β-relaxation, probably due to local rotations of the terminal groups or other side chains of gluten or starch [9]. The Tβ of the dough without freezing at −18 °C with calcium carbonate (CA-In) were lower than those of the other calcium salts. After frozen storage, the Tβ of wheat dough (C) and doughs with carbonate (CA, CA-In) and citrate (CI) increased, except for doughs with LA and CI-In, which did not change significantly.

### 3.3. Optimization of Fermentation Times of Frozen Dough and Bakery Quality of Breads

**Figure 5:**
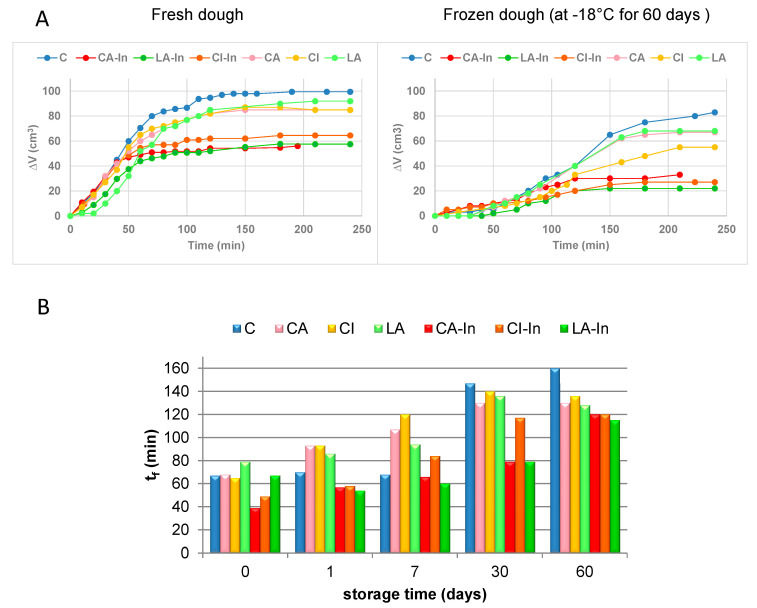
Fermentation of dough. (**A**). Fermentation curves obtained from fresh dough and frozen dough at −18 °C for 60 days, (**B**) fermentation time of frozen dough at −18 °C for 0, 1, 7, 30 and 60 days.

Figure 5A shows the change in volume (ΔV) as a function of time for fresh and frozen dough for 60 days at −18 °C. The increase in ∆V of fresh dough during fermentation was lower in doughs with inulin, except for the control (C), which presented the greatest expansion of the dough. A similar trend was observed in the doughs previously frozen for 60 days; however, for the same formulation, the Vmax reached was lower than in the fresh doughs and was achieved in a more slowly form (Figure 5A). This behavior could negatively influence the bread volume.

Figure 5B shows the optimal fermentation times (tf) obtained from the doughs previously frozen for 0, 1, 3, 7 and 60 days. In all formulations, the tf of thawed dough after they were frozen and stored at −18 °C increased with the time. This can be attributed to a lower production of CO_2_ resulting from a lower number of active yeasts and/or from a decrease in the ability of gluten to retain it [31,32]. Metabolites produced during yeast fermentation can alter the stability of frozen products. During storage, yeasts in frozen doughs can be affected by partially losing their fermentation capacity because of the physiological effects of frozen water on cells or of the concentration of certain fermentation metabolites [32].

### 3.4. Quality of Bread Made from Frozen Dough

Specific volume (Vs) and crust color of breads and also moisture, firmness, springiness, and alveoli distribution of crumbs were evaluated as parameters of bakery quality of breads made from previously frozen dough.

Values of bread Vs are shown in Table 3. For different formulations of the same day, the Vs values of breads with In and Ca were lower than in the absence of In, especially with organic calcium salts. Up to day seven of dough freezing, no significant differences in Vs were found (data not shown). At the end of the storage (60 days), the Vs of bread with carbonate (CA) did not significantly change with respect to wheat bread (C), whereas in the presence of inulin (CA-In), the Vs decreased by 25% with respect to that obtained in samples from unfrozen dough. After 60 days of storage, lower values of Vs were found in breads prepared with inulin and calcium (especially in those with carbonate and citrate), decreasing by 28% and 18% in Ca-In and CI-In breads, respectively. In contrast, in LA-In breads the Vs decreased by only 12% as a consequence of freezing, due to the protective effect of inulin and the destabilizing effect of the lactate anion on the gluten network.

Filipović et al. [32] studied the effect of different commercial fibers (5%) on frozen dough (−18 °C for 60 days) used for breadmaking. They found a protective effect of inulin, obtaining an improvement in bread quality. Nevertheless, when using fructo-oligosaccharides of low DP, these molecules were included in the gluten structure, contributing to deteriorate the matrix and leading to a depreciation of the dough rheological properties during fermentation, and also with an adverse effect on bread volume and crumb quality.

The crusts of breads prepared with calcium and inulin from unfrozen doughs were browner than those of breads prepared without the prebiotic (C, CA; CI, LA) (Table 3). After storage, all breads made from frozen doughs significantly increased the BI in all formulations. This increase could be due to the fact that during freezing the structure may break down because of the ice formation (e.g., proteins, releasing free amino groups), thus favoring the Maillard reaction.

The crumb of highest moisture was that of wheat bread (C) (Table 3). Breads with calcium salts retained a smaller amount of water. In a different way to dough (Figure 1A), calcium salts were not able to retain moisture in crumbs during baking, possibly because they interfere with water-protein interactions in gluten. This low moisture of crumbs from freezing dough would be responsible for a high firmness, considering that Vs did not change.

The firmness of crumb is one of the parameters that is directly related with the perception of freshness in bread by consumers [33]. Firmness increased with dough storage time (Table 3). Several authors found similar results, and they attributed the increase of firmness to the loss of water during the frozen storage of dough at −18 °C [34,35,36].

In general, the crumb firmness of breads containing different calcium salts, with and without inulin, increased with 60 days of dough storage in a frozen state (Table 3). In the presence of inulin, an inverse relationship between firmness and Vs was observed; therefore, an increase of crumb firmness would probably be related with a decrease in Vs. After 60 days, the firmness of breads with calcium did not significantly differ from the control sample (wheat bread). For breads with CaCO_3_ and CaLA_2_ and inulin (CA-In, LA-In), this behavior was intensified. Inulin reinforces the gluten network, leading to more elastic doughs, albeit ones that form breads of less volume with firm crumbs.

The springiness of crumbs of unfrosted dough was similar to that of frozen dough (Table 3). Nevertheless, for crumbs containing calcium and inulin, springiness decreased with dough storage (~5%), with the exception of CI-In sample, which decreased 11%.

**Figure 6:**
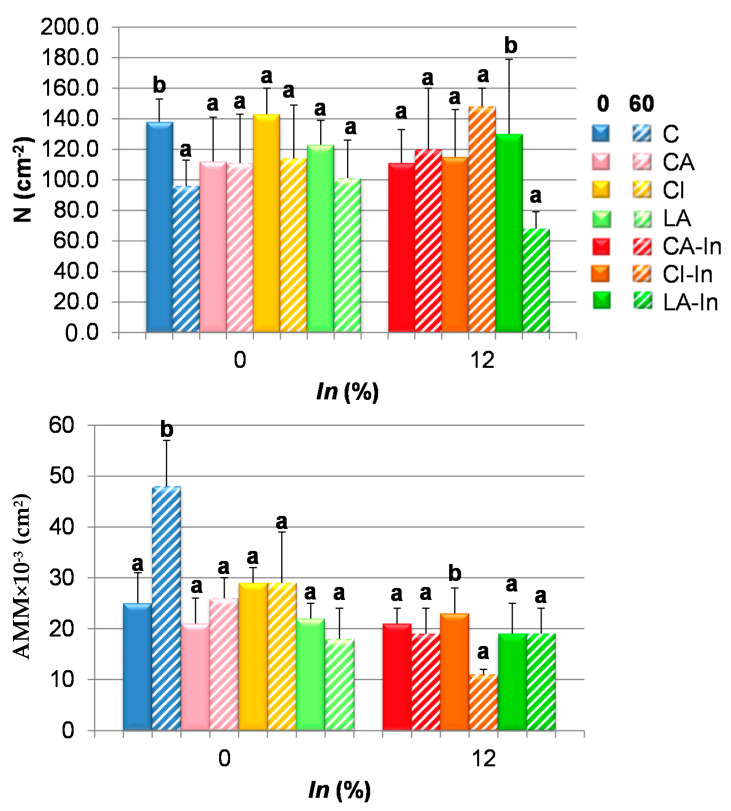

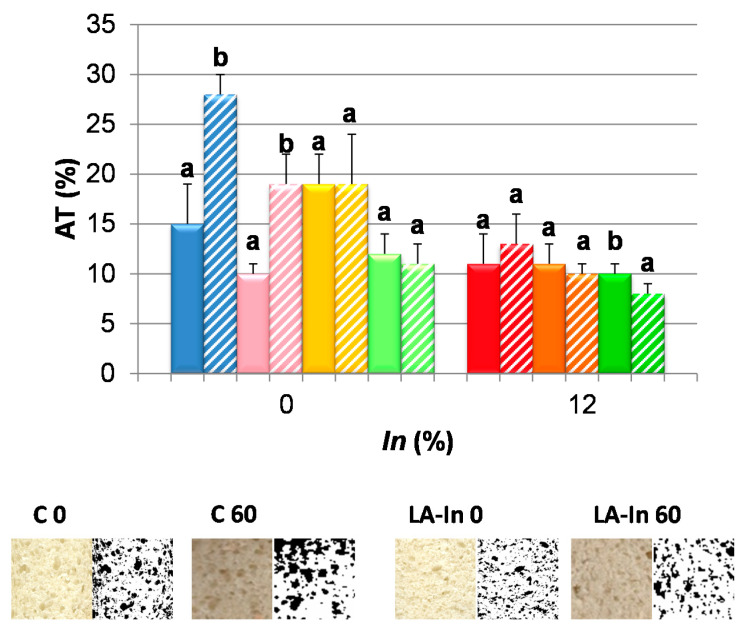
Crumb alveolate parameters: number of alveoli per area (**N**), mean area of alveoli (**AMM**) and air fraction (**AT**) of fresh and stored doughs at −18 °C for 60 days. Different letters in the same formulation indicate significant differences (*p* < 0.05). Bars: mean± standard deviation. Crumb and binary images of C0, C60, LA-In 0 and LA-In60 breads.

The effect of frozen storage of dough in the alveoli composition of crumb was studied by image analysis. Frozen dough changed the number of alveoli in crumb (N); C and LA-In (Figure 6), mainly LA-In formulations presented 48% less alveoli than crumbs of fresh dough (LA). After 60 days of freeze dough storage, the crumb of wheat bread presented a significant increase of the average area of the alveolus (AMM) (Figure 6) jointly with a decrease in N. These results suggest that crumb from stored dough contained less alveoli, but of significant size, indicating a more open crumb structure, probably due to a mechanic damage of gluten matrix as a consequence of ice crystals formed in dough during freezing. Scanning Electron Microscopy (SEM) allowed the confirmation of a damaging effect of ice crystals in non-fermented dough stored in the frozen state [4].

Control crumb presented a major amount of air (Figure 6, AT) and resulted in a high moisture; therefore, it is supposed to be softer, an effect that was not demonstrated. The structure of crumb is more aerated. CA crumbs arising from frozen dough presented higher AT, while crumbs from LA-In showed a lower AT and higher firmness. The images of crumbs of wheat bread (C) and with calcium lactate and inulin (LA-In) can be observed at the bottom of Figure 6. Samples containing calcium lactate were selected because the porosity of these crumbs changed with freezing dough.

**Figure 7:**
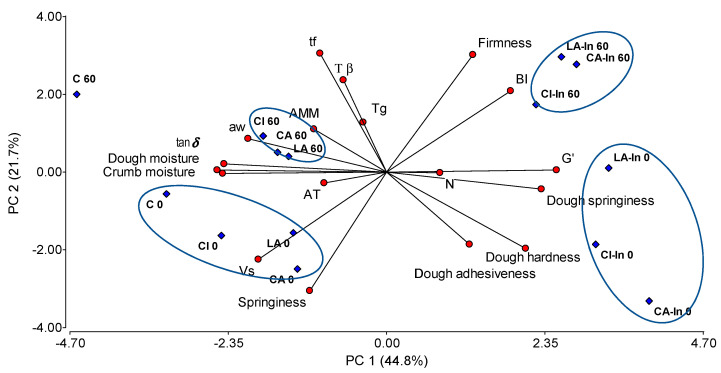
Biplot (PCA) of correlation between fresh and stored doughs at −18 °C for 60 days and its bread quality.

PCA performed on fresh and frozen stored doughs explained 66.5% of the total variance (Figure 7). Principal component 1 (PC1) accounted 44.8% of the total variance and was associated to moisture, aw, tan δ and in the opposite direction hardness, elasticity and G′. On the other hand, PC2 was mainly related to baking quality parameters, such as fermentation time, browning index, firmness and the elasticity of crumbs, and also dough hardness and Tβ. In addition, samples were grouped according to the content or absence of inulin (0, In) and the freezing time (0, 60 days), and were independent of the type of calcium salt.

Dough without inulin were the wettest and least elastic (located close to the tan δ vector and away from the dough springiness vector). On the other hand, dough with inulin were the hardest and most elastic ones, and had the lowest moisture and water availability (aw) regardless of freezing time. With respect to PC2, there was a clear effect of freezing, the frozen doughs required longer fermentation times (tf), and were softer and more adhesive than the unfrozen ones; the breads were browner, with firmer crumb and less elastic than those made from fresh dough. We can conclude that inulin is a very important ingredient in protecting the dough during freezing despite the less desirable effects in the baked products. The effect of the type of calcium salt used is more important in fresh doughs than in frozen doughs, as can be seen from the greater dispersion of the points in the quadrant.

## 4. Conclusions

The rheological and thermomechanical properties of the dough and the baking quality of the breads were affected by the presence of prebiotic and calcium during freezing. Moisture did not change after freezing the dough for 60 days in the presence of inulin. The hardness and adhesiveness of wheat dough and dough with calcium and inulin decreased after 60 days of storage. The viscoelastic behavior of all of the doughs was not affected during storage under freezing conditions. In doughs with calcium carbonate, with and without inulin, Tg increased, an effect associated with an increase in the glassy state of the matrix with freezing; while doughs with organic calcium salts and inulin decreased Tg, which is associated with a softer structure. Therefore, the type of anion affected the gluten matrix (e.g., interactions between polymers, the inulin and these salts, and their capacity of holding water during freezing). The fermentation times of all formulations increased during storage and the thawed dough expanded less during fermentation, especially in the presence of inulin.

Regarding bread quality, in the absence of inulin and in the presence of all calcium salts, Vs did not change, although the crumb retained less water and thus was harder. The breads containing inulin presented moister crumbs, although they were much firmer after storing the dough in the frozen state; this effect corresponds to lower Vs. The excessive increase in firmness of the LA-In crumb could be due to a decrease in the proportion of air retained in the alveoli, since the specific volume was slightly affected.

Among all the investigated formulations, breads including calcium salts that stabilized the gluten network in the presence of inulin (CI-In and CA-In) presented lower Vs, greater firmness and similar alveoli composition; this was in contrast to the breads containing inulin in the absence of calcium (LA-In) in which the Vs was not modified but the crumbs were very firm and with little air retained. Therefore, the dough freezing technology would be useful to apply to doughs with carbonate and mainly with citrate calcium, since the latter salt produced a smaller increase in firmness compared to the inorganic salt.

## Figures and Tables

**Table 1:** Formulations and farinographic parameters of blend.

Formulation	Wheat Flour (g)	Calcium (ppm)	Inulin * (%)	W_abs_ (%)	t_d_ (min)
**C**	100	0	0	56.0	12
**CA**	100	1800	0	53.6	20
**CI**	100	1800	0	53.7	24
**LA**	100	1800	0	53.3	11
**CA-In**	100	2400	12	51.0	22
**CI-In**	100	2400	12	52.5	33
**LA-In**	100	2400	12	52.6	29

C: Control, different calcium salts (carbonate—CA, citrate—CI and lactate—LA) and blends with calcium and inulin (CA-In, CI-In, LA-In). W_abs_: water absorption. t_d_: development time. * Inulin: synergy1^®^.

**Table 2:** Glass transition temperature (Tg) and β-relaxation obtained from loss modulus curves at 1 Hz.

Formulation	Time of Dough Freezing (days)	Tg (°C)	Tβ (°C)
**C**	0	−39.2 ± 0.2 cd	−63.8 ± 0.8 bcde
	60	−43.6 ± 1.5 bc	−58.4 ± 1.1 f
**CA**	0	−54.9 ± 2.3 a	−84.6 ± 1.8 a
	60	−32.3 ± 3.7 e	−66.2 ± 0.2 bcd
**CI**	0	−41.8 ± 2.0 bc	−65.8 ± 0.1 bcd
	60	−39.0 ± 0.2 cd	−59.4 ± 3.6 ef
**LA**	0	−40.2 ± 0.9 bc	−64.4 ± 1.8 bcde
	60	−31.2 ± 2.3 e	−66.8 ± 4.6 bc
**CA-In**	0	−51.6 ± 0.0 a	−83.6 ± 0.1 a
	60	−39.2 ± 2.7 cd	−62.1 ± 3.3 cdef
**CI-In**	0	−38.9 ± 5.4 cd	−65.3 ± 5.9 bcd
	60	−45.3 ± 4.6 b	−60.6 ± 0.9 def
**LA-In**	0	−34.4 ± 1.1 de	−62.9 ± 1.1 cdef
	60	−44.3 ± 1.2 bc	−69.4 ± 4.4 b

Different letters in the same column indicate statistical differences (*p* < 0.05).

**Table 3:** Bakery quality of breads prepared with doughs frozen at −18 °C for 0 and 60 days.

	Ca (ppm)	In (%)	Time Frozen Dough (days)	Vs (cm^3^/g)	Crust	Crumb		
					BI	Moisture (%)	Firmness (N)	Springiness
**C**	0	0	0	2.6 ± 0.2 e	41 ± 6 b	43.7 ± 0.4 h	10 ±2 b	0.96 ± 0.02 de
			60	2.6 ± 0.1 de	56 ± 8 d	44.6 ± 0.2 i	15 ± 2 c	0.93 ± 0.02 cd
**CA**	1800	0	0	2.6 ± 0.1 de	55 ± 7 d	42.2 ± 0.1 f	8.7 ± 0.5 ab	1.02 ± 0.09 f
			60	2.6 ± 0.2 de	83 ± 9 g	41.2 ± 0.1 e	13 ± 2 c	0.94 ± 0.02 cd
**CI**	1800	0	0	2.8 ± 0.3 e	35 ± 5 a	42.9 ± 0.0 g	7.0 ± 0.8 a	0.99 ± 0.01 f
			60	2.5 ± 0.1 d	76 ± 5 f	41.0 ± 0.1 d	13 ± 2 c	0.94 ± 0.02 cd
**LA**	1800	0	0	2.8 ± 0.2 e	48 ± 11 c	42.3 ± 0.2 f	7.8 ± 0.4 a	0.99 ± 0.04 ef
			60	2.5 ± 0.2 d	57 ± 11 d	41.5 ± 0.1 e	13 ± 2 c	0.94 ± 0.02 cd
**CA-In**	2400	12	0	2.5 ± 0.1 de	97 ± 9 h	39.1 ± 0.4 a	10.7 ± 0.2 b	0.94 ± 0.01 cd
			60	1.9 ± 0.1 ab	121 ± 9 j	39.7 ± 0.4 b	28 ± 4 e	0.88 ± 0.03 ab
**CI-In**	2400	12	0	2.2 ± 0.2 c	71 ± 9 e	39.0 ± 0.2 a	12.7 ± 0.9 a	0.99 ± 0.01 f
			60	1.8 ± 0.2 a	108 ± 10 i	39.8 ± 0.1 b	17 ± 2 d	0.88 ± 0.03 ab
**LA-In**	2400	12	0	2.1 ± 0.2 bc	76 ± 18 f	39.7 ± 0.1 b	16.4 ± 3.4 d	0.91 ± 0.02 bc
			60	1.8 ± 0.3 a	141 ± 9 k	40.2 ± 0.1 c	27 ± 4 e	0.86 ± 0.04 a

Vs: Specific volume; BI: browning index. Values: mean ± standard deviation. Different letters in the same column indicate significant differences (*p* < 0.05).

## Data Availability

The data supporting the results of this study are included in the present article.
